# Impact of Skin Tissue Collection Method on Downstream MALDI-Imaging

**DOI:** 10.3390/metabo12060497

**Published:** 2022-05-30

**Authors:** Manoj Yadav, Prem Prashant Chaudhary, Brandon N. D’Souza, Jacquelyn Spathies, Ian A. Myles

**Affiliations:** Epithelial Therapeutics Unit, National Institute of Allergy and Infectious Disease, National Institutes of Health, Bethesda, MD 20892, USA; premprashant.chaudhary@nih.gov (P.P.C.); dsouza.80@buckeyemail.osu.edu (B.N.D.); jacquelyn.spathies@nih.gov (J.S.); mylesi@niaid.nih.gov (I.A.M.)

**Keywords:** MALDI-MSI, FFPE, IHC, metabolites

## Abstract

MALDI imaging is a novel technique with which to study the pathophysiologies of diseases. Advancements in the field of metabolomics and lipidomics have been instrumental in mapping the signaling pathways involved in various diseases, such as cancer and neurodegenerative diseases (Parkinson’s). MALDI imaging is flexible and can handle many sample types. Researchers primarily use either formalin-fixed paraffin-embedded (FFPE) or fresh frozen tissue samples to answer their scientific questions. FFPE samples allow for easy long-term storage, but the requirement for extensive sample processing may limit the ability to provide a clear picture of metabolite distribution in biological tissue. Frozen samples require less handling, but present logistical challenges for collection and storage. A few studies, mostly focused on cancer cell lines, have directly compared the results of MALDI imaging using these two tissue fixation approaches. Herein, we directly compared FFPE and fresh frozen sample preparation for murine skin samples, and performed detailed pathway analysis to understand how differences in processing impact MALDI results from otherwise identical tissues. Our results indicate that FFPE and fresh frozen methods differ significantly in the putative identified metabolite content and distribution. The fixation methods shared only 2037 metabolites in positive mode and only 4079 metabolites in negative ion mode. However, both fixation approaches allowed for downstream fluorescent staining, which may save time and resources for samples that are clinically precious. This work represents a direct comparison of the impacts of the two main tissue processing methods on subsequent MALDI-MSI. While our results are similar to previous work in cancer tissue, they provide novel insights for those using MALDI-MSI in skin.

## 1. Introduction

Matrix-assisted laser desorption ionization (MALDI) mass spectrometry imaging (MSI) is an expanding tool which aims to understand the presence and distribution of different biomarkers, such as peptides, lipids, and other small metabolites. Unlike traditional MS, the spatial distribution of metabolites in tissue section can be studied using the MALDI-MSI. However, tissue samples must first be preserved prior to analysis. Two types of preservation methods are widely used: formalin-fixed paraffin-embedded (FFPE) and fresh frozen. FFPE preservation allows for retrospective analysis of tissue samples even after long term storage, but requires the samples to pass through enzymatic digestion, dehydrations, rehydrations, and antigen retrieval protocols. Frozen samples do not require enzymatic processing, but are reliant on immediate freezing and proper long term storage to preserve sample integrity [1,2,3].

A limited number of reports have directly compared FFPE and fresh frozen preservation techniques on subsequent putative metabolite identification. These reports are from prostate cancer, colon cancer, and mouse models of Parkinson’s disease [1,2,3,4,5]. Specifically, prior studies have evaluated prostate cancer cell lines (LNCaP and LNCaP-Abl [1], Vero-B4 cells, LLC-PK1 cells), human colorectal cancer cell lines (DLD-1, HCT 116 [6]), murine melanoma (B16-F0 and B164A5), human melanoma (A375), human breast carcinoma (MCF7 and MDA-MB-231), human liver carcinoma (HepG2) [7], murine fallopian tube cells, and high-grade ovarian carcinoma cell lines [8]. Currently, however, no information on preservation selection is available for skin tissue. In this study, we show the comparative metabolic profiles of skin tissue samples in FFPE and fresh frozen skin tissue via both positive and negative ion modes. This study elucidates collection and processing methods for skin tissue samples that may enable researchers to better determine which method is optimal for their research. Skin diseases are a complex set of events influenced by intracellular and extracellular molecules. Here we report the major pathway differences between murine skin tissue from the same animal. This analysis is particularly useful for the research community working on skin diseases.

## 2. Results

### 2.1. MALDI Positive Ion Mode Imaging of FFPE and Fresh Frozen Skin Tissue Samples

**Figure 1:**
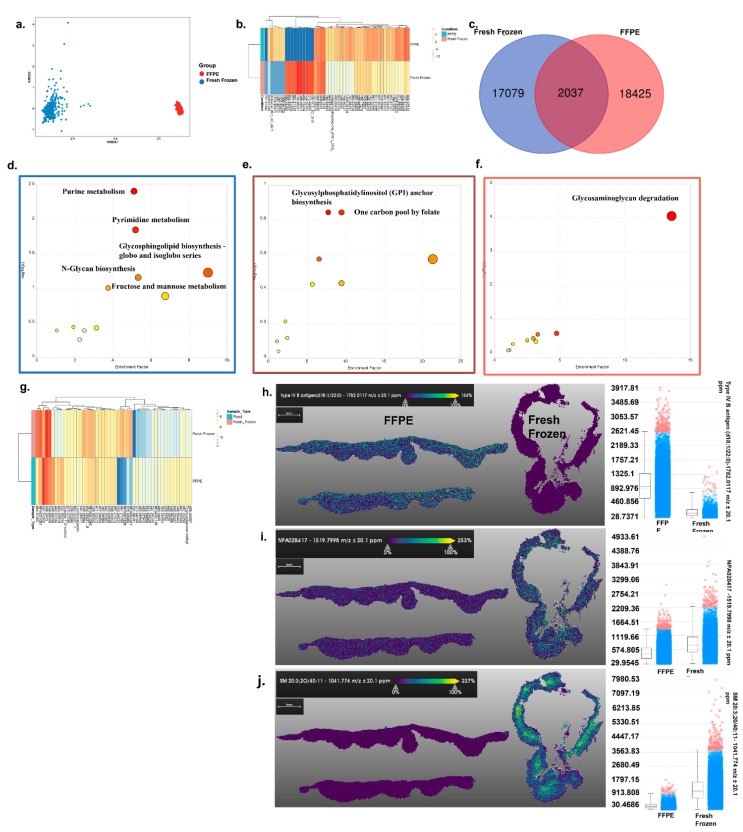
MALDI positive mode imaging of skin tissue: (**a**) Nonmetric dimensional plot of the metabolites present in fresh frozen and FFPE samples. An ANOSIM test showed that there are significant differences in the metabolic profiles of the groups tested. (Stress: 0.03219174, ANOSIM statistic R: 1, significance: 1 × 10^−4^). (**b**) Heatmap plot of the top 50 most significantly different metabolites between the two groups. (**c**) Venn diagram showing the unique and shared metabolites from the two groups. (**d**–**f**) Metabolic pathway analysis plot created using MetaboAnalyst 5.0, USA. Plots depict several metabolic pathways that differ between the fresh frozen and FFPE samples tested. (**d**) Differential metabolites calculated from FFPE vs. fresh frozen samples. (**e**) Common metabolites from the FFPE and fresh frozen groups were sorted based on *p*-values (high to low). (**f**) Differential metabolites were calculated from fresh frozen vs. FFPE. (Box color corresponds to the same color from the Venn diagram). The *y*-axis is the log scale of the *p*-value; pathways that were most significantly different are characterized by high –log10(*p*) values. (**g**) The top 50 common metabolites were sorted based on *p*-value and are represented as a heatmap with their detected intensities. (**h**–**j**) Specific metabolite distribution images are presented along with an intensity box plot. (**h**) An image of type IV B antigen distribution in both tissues and an intensity box plot of the same metabolite’s distribution. (**i**) An image of NPA028417 distribution in both tissues and an intensity box plot of the same metabolite’s distribution. (**j**) An image of SM 20:3: 20/40 distribution in both tissues and an intensity box plot of the same metabolite’s distribution.

For comparative MALDI imaging, tissue samples were collected from the same mice, from the same area of skin, at the same time: we divided the tissue in half. Comparative analysis of FFPE and fresh frozen skin tissue samples imaged with positive ion mode revealed significant differences in the presence and distribution of metabolites. NMDS plot analysis using a Bray–Curtis dissimilatory matrix between FFPE and fresh frozen skin tissue showed that metabolites detected in the samples were different, despite the samples being from the same mice (ANOSIM *p* = 1 × 10^−4^) (Figure 1a). The top 50 metabolites were chosen based on *p*-value (FDR) and represented as a heatmap plot based on the intensity of the metabolite’s peak (Figure 1b). The heatmap analysis revealed the metabolite at *m*/*z* of 629.01196 was present in both the samples but differed in the signal intensity (Figure 1b). In total, 2037 metabolites were detected in both sample preservation formats, 17,079 were detected only in fresh frozen, and 18,425 were detected only in FFPE preserved tissue (Figure 1c).

Pathway analysis in positive ion mode revealed that FFPE preservation led to significant increases in the detection of putative metabolites associated with purine metabolism, pyrimidine metabolism, glycosphingolipid biosynthesis—globo and isoglobo series, and N-glycan biosynthesis (Figure 1d and Supplementary Table S1). In contrast, fresh frozen only led to stronger detection of the glycosaminoglycan degradation pathway (Figure 1f and Supplementary Table S2). Evaluating the 2037 common metabolites by pathway analysis putatively identified only significant enrichment of xenobiotic metabolism by cytochrome P450 (Figure 1e and Supplementary Table S3). However, while these metabolites were putatively identified in both tissue preservation formats, differential intensity signals were still observed (Figure 1g). Furthermore, the distributions of the specific annotated metabolites were different for the preservation methods. For example, type IV B antigen intensity was higher in FFPE (Figure 1h), SM 20:3: 20/40 signal was more abundant in fresh frozen tissue, (Figure 1i), and NPA028417 was found to be near equal in both tissues (Figure 1j). Only fresh frozen tissue demonstrated metabolite distribution in the tissue that was consistent with the expected histology of the skin (Figure 1h–j).

### 2.2. MALDI Negative Ion Mode Imaging of FFPE and Fresh Frozen Skin Tissue Samples

**Figure 2:**
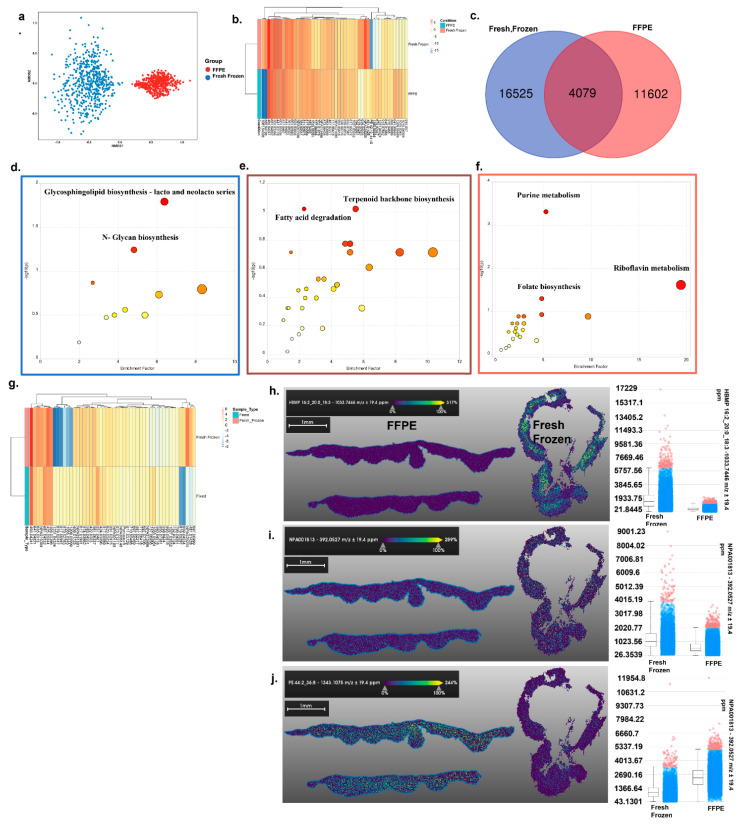
MALDI negative mode imaging of skin tissue: (**a**) Nonmetric dimensional plot of the metabolites present in fresh frozen and FFPE samples. Significant *p*-values from ANOSIM test showed there are differences in the metabolic profiles of the group tested (Stress: 0.03219174, ANOSIM statistic R: 1, significance: 1 × 10^−4^). (**b**) The heatmap plot of the top 50 most significantly different metabolites between the two groups. (**c**) Venn diagram showing the unique and shared putative metabolites between the two comparisons. (**d**–**f**) Metabolic pathway analysis plot created using MetaboAnalyst 5.0. Plots depict several metabolic pathway alterations when unique and shared metabolites from the fresh frozen and FFPE samples were tested. (**d**) Differential metabolites calculated from FFPE vs. fresh frozen. (**e**) Common metabolites from the Venn diagram were sorted based on *p*-value (high to low). (**f**) Differential metabolites calculated from fresh frozen vs. FFPE. (Pathway graph color box corresponds to the same color in Venn diagram). The *y*-axis is the –log10 of the *p*-value; pathways that were most significantly changed are characterized by high –log10(*p*) values (top middle or right region). (**g**) The top 50 common metabolites were sorted based on *p*-value and represented as a heatmap. (**h**–**j**) Specific metabolite distribution images are presented along with an intensity box plot. (**h**) HBMP 16:2_20:0_18:3 distribution in both tissues and an intensity box plot of the same metabolite’s distribution. (**i**) NPA001813 distribution in both tissues and an intensity box plot of the same metabolite’s distribution. (**j**) PE 44:2_36:8 distribution in both tissues and an intensity box plot of the same metabolite’s distribution.

We repeated this analysis using the negative ion mode of MALDI-MSI. Like positive ion mode imaging, comparative NMDS plots showed stark differences in metabolite presence between sample collection methods (ANOSIM *p* = 1 × 10^−4^) (Figure 2a) and differential intensities in top metabolites (Figure 2b). In total, 4079 common putative metabolites were detected in both tissue preservation methods, 16,525 were unique to fresh frozen, and 11,602 were unique to FFPE (Figure 2c).

Pathway analysis of metabolites that were better putatively identified by FFPE processing identified enrichment of glycosphingolipid biosynthesis—lacto and neolacto series, and N-glycan biosynthesis pathways (Figure 2d and Supplementary Table S4). In contrast, fresh frozen tissue better putatively identified metabolites associated with the purine metabolism, riboflavin metabolism, and folate biosynthesis pathways (Figure 2f and Supplementary Table S5). Pathways putatively identified in both tissue storage types included steroid hormone biosynthesis and riboflavin metabolism (Figure 2e and Supplementary Table S6). However, differential intensities for the top metabolites were found (Figure 2g). Similarly to positive ion mode, intensity differences of specific metabolites could be putatively identified (HBMP 16:2_20:0_18:3 more abundant in the fresh frozen tissue (Figure 2h), PE 44:2_36:8 higher in FFPE (Figure 2i), and NPA001813 equal in both (Figure 2j) Additionally, similarly to positive ion mode, only fresh frozen processing provided a useful spatial distribution (Figure 2h–j).

### 2.3. Spatial Distribution of Metabolites in Skin Tissue via MALD-MSI Combined with Immunohistochemistry

**Figure 3:**
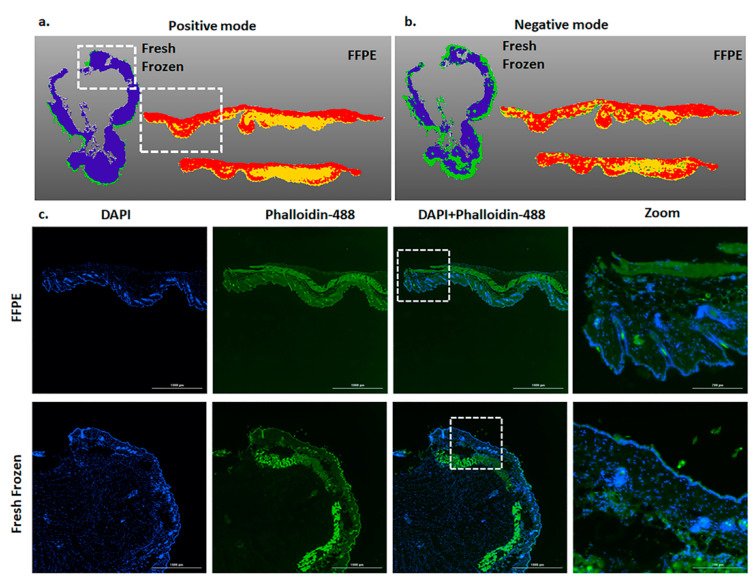
Segmentation and Immunohistochemistry of skin tissue post-MALDI Imaging: (**a**,**b**) Representative images show the differential presence of metabolites from the skin tissue in FFPE and fresh frozen conditions in positive and negative mode MALDI imaging, respectively. (White dotted box represents the tissue area imaged in ©). (**c**) Representative immunohistochemistry images show the FFPE and fresh frozen tissue sections stained with Phalloidin-488 (green staining actin) and DAPI (blue staining nucleus) stains. The white dotted box shows the zoomed image.

To further investigate the spatial distributions of metabolites in FFPE and fresh frozen tissue samples after MALDI imaging, we performed segmentation using SCiLS software and found that both tissues varied in terms of overall metabolite distribution (Figure 3a,b). Segmentation groups areas by metabolic similarity and displays them as shared colors overlayed on the tissue.

Previously, Spraggins et al., 2019 showed that cancer tissue that has undergone MALDI-MSI can be used for the H and E staining [1,9]. Given the limited availability of many clinically relevant samples, we further tested if skin samples could be processed for immunohistochemistry staining post MALDI-MSI. Both Phalloidin and DAPI demonstrated equivalent and robust staining in both tissue types after MALDI-MSI aquation (Figure 3c).

## 3. Discussion

Previous studies have compared MALDI imaging of FFPE and fresh frozen tissue [10,11]. This analysis represents a direct comparison of the impacts of the two main tissue processing methods on subsequent MALDI-MSI using skin tissue. The unique lipid content and distribution of skin tissue presents a distinct challenge for untargeted metabolomic assessment using MALDI-MSI. Putative identification of metabolite distribution by spatial maps may be as beneficial for studying skin pathophysiology, as it has been for other tissues [2,4,9].

Our data indicate that putative metabolite detection can be heavily influenced by the researcher’s selection of ion mode and preservation technique. We hope our data can assist others using MALDI-MSI on skin tissue with both their planning of future experiments and interpretation of experimental results. For example, FFPE may be optimal for researchers investigating glycosphingolipid biosynthesis (globo and isoglobo series), or N-glycan biosynthesis regardless of ion mode. Investigation into pyrimidine metabolism may be optimal with FFPE tissue storage and positive ion mode, whereas purine metabolism may be more readily detected in fresh frozen tissue using negative ion mode. Fresh frozen, negative ion mode may also be ideal for riboflavin metabolism and folate biosynthesis, whereas fresh frozen tissue should be run with positive ion mode to optimize evaluation of the glycosaminoglycan degradation pathway. However, post-MALDI-MSI, all tissue samples can be further processed with IHC validation or H&E staining [1,9].

Our ion distribution results indicate that FFPE tissue offers very poor spatial resolution compared to fresh frozen tissue. This anticipated finding may be critical for localizing a specific metabolite to the epidermis or dermis but may be unnecessary if specific spatial distribution is not important, as may be the case if only the presence or absence of a specific biomarker is in question. Similarly, frozen tissue offers high spatial resolution under both positive and negative ion modes. This was further confirmed by our segmentation images of both tissues.

Our study was limited to murine skin samples and did not assess metabolites over 2500 *m*/*z*. Furthermore, FFPE samples, due to a large number of intermediate dehydration, rehydration, and antigen retrieval steps under high-pressure tissue sections, are prone to dislodge from the slide, which may contribute to variability in the results. In conclusion, we hope this study assists researchers in selecting which tissue preservation and MALDI acquisition methods will be most likely to answer their specific scientific questions. Furthermore, our findings can inform future studies related to skin diseases through helping reviewers contrast conclusions with the author’s methodology.

## 4. Material and Methods

### 4.1. Mice

BalbC mice, male and female (*n* = 3 each case), age 6–10 weeks, were used in this study. All experiments were approved and monitored under approved protocols from the National Institute of Allergy and Infectious Diseases (NIAID). For FFPE and fresh frozen comparison, tissues were collected from same mice from one area of skin and then split in half for comparative analysis.

### 4.2. FFPE Tissue

**Figure 4:**
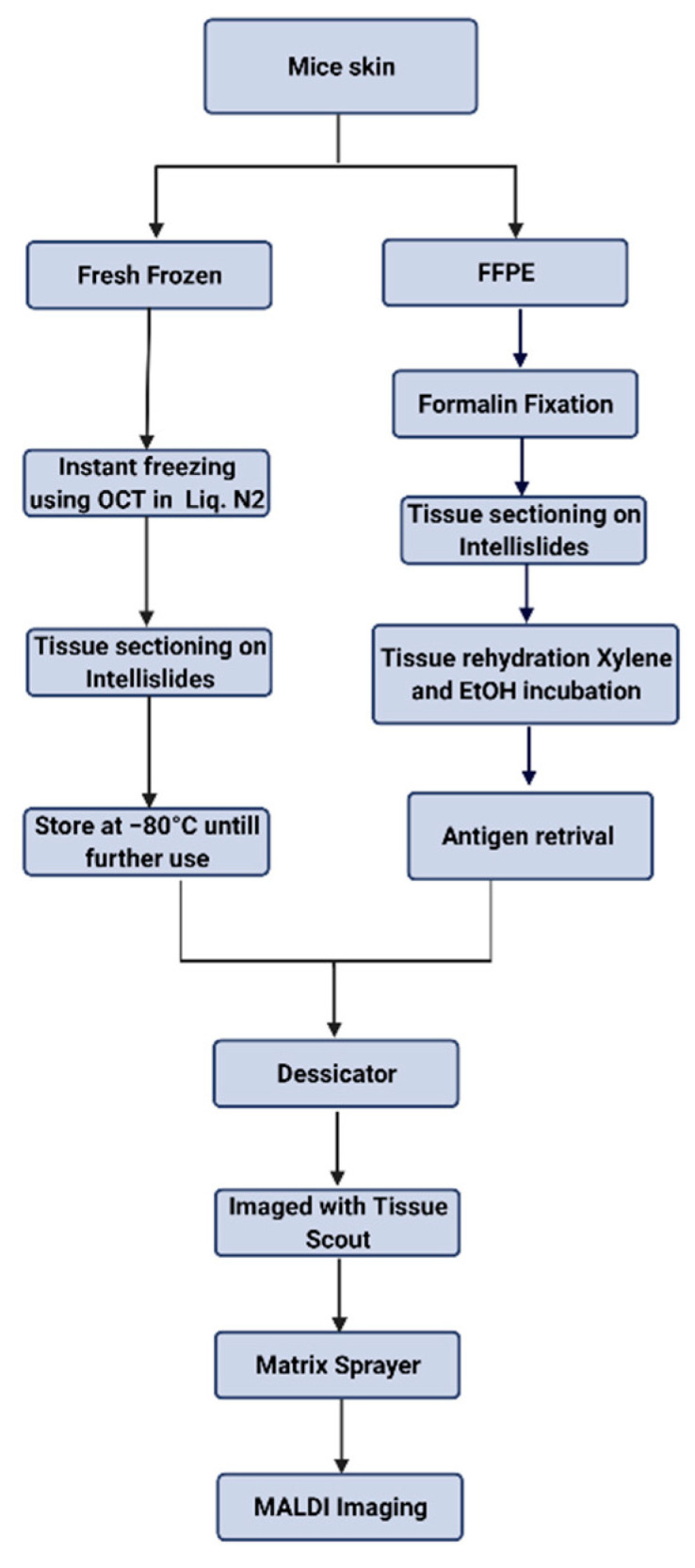
Schematic representation of sample processing.

After collecting the mouse skin, tissue samples were immediately placed in a formalin solution for 72 h at room temperature. Samples were then transferred to a 70% ethanol solution and embedded in paraffin; tissue was sectioned to a thickness of 10 µm and placed on Intellislides (#1868957 Bruker Scientific LLC, Billerica, MA, USA). To rehydrate the tissue section, slides were incubated in different concentrations of dehydrant as follows: twice in Xylene (# 23044192 Fisher Scientific Waltham, MA, USA) for 10 min each, and then in 100% ethanol twice for 10 min each, 95% ethanol for 5 min, 70% ethanol for 5 min, and 50% ethanol for 5 min. Tissue sections were rinsed in water and placed in 1× PBS for 10 min. For antigen retrieval, tissue slides were placed in an instant pot under high pressure for 40 min in PH = 6 epitope retrieval solution (# RE 7113 Leica Biosystems, IL, USA). Tissue slides were then rinsed in distilled water and placed in PBS (#10010023 Thermo Fisher Scientific, Waltham, MA, USA). Then slides were placed in a desiccator and imaged with Tissue Scout (Bruker) before proceeding to the matrix sprayer. Schematic representation of all the steps has been shown in Figure 4. 

### 4.3. Fresh Frozen Tissue

The tissue skin biopsy samples were collected in a Tissue-Tek Cryomold (#4457 Sakura, CA, USA). The embedding medium used was OCT Compound Tissue Plus (#4585 Scigen Scientific Gardena, CA, USA). Tissue was placed on the embedding medium; subsequently, extra OCT was layered on top of the tissue. The sample in cryomold was then placed in a liquid nitrogen tank for 2 h and transferred to a −80 °C freezer until samples were sectioned. After paraffin embedding, tissue was sectioned to a thickness of 10 µm, placed on Intellislides (#1868957 Bruker Scientific LLC, Billerica, MA, USA), and stored at −80 °C until imaging.

### 4.4. Matrix Sprayer

FFPE and fresh frozen tissue slides were placed in a desiccator and imaged with Tissue Scout (Bruker) after drying. Tissue sections were sprayed using the HTX TM-Sprayer (HTX Technologies). The matrix solution used was 20 mg/mL 5-dihydroxybenzoic acid (#149357-20G Sigma-Aldrich Inc, Missouri, USA) in 100% acetone and 0.1% trifluoroacetic acid(#302031 Sigma). The following conditions were used to spray all the slides: nozzle temperature of 50 °C, plate rate of 0.100 mL/min, Z arm velocity of 1200, 14 passes, moving pattern CC, and track spacing of 2.

### 4.5. Data Acquisition

After spraying the slides with matrix solution, slides were loaded in a slide holder from Bruker, and we ran the slides in timsTOF flex (Bruker). Slides were run in both positive and negative ion modes to facilitate comparison. The tissue was scanned with both MS and TIMS settings at a resolution of 20 µm. The MS settings were: scan range 20–2500 *m*/*z* in positive MS scan mode. The TIMS settings were: 1/K0 0–8 − 1.89 V×s/cm^2^, ramp time of 200 ms, acquisition time of 20 ms, duty cycle = −10%, and ramp rate of 4.85 Hz.

### 4.6. Data Processing and Metabolite Annotations

Acquired raw data were initially processed with SCiLS lab 2021a (Bruker Scientific LLC, Billerica, MA, USA). Sample preparation, acquisition parameters, and analysis settings followed guidelines from Sumner et al., 2007 [12]. To remove the background noise, we performed the following steps: after assigning region (whole tissue), peaks were moved to local maxima, and we selected strong denoising as an option and normalized the data with total ion count. Data were then saved as “peak list.” The mass range for assigning the regions (entire tissue section) was 20–2000 *m*/*z*. For segmentation in the SCiLS, we selected peak list and moved the sliding window until we got maximum intervals. In this step, we selected an intensity threshold of 7242.1791 and it gave us 9812 intervals. This step further helped with removing noise and intensity peaks below 7242.1791. We then normalized the data by root mean square. We created regions out of desired segments and repeated this until all the regions were created.

After assigning regions (entire tissue section) to SCiLS regions, the file was exported to Metaboscape 2021b (Bruker, USA) for annotations and further downstream analysis. In Metaboscape, ROI subsampling parameters were as follows: width—5, height—5, maximum number of speckles/ROI—500, and intensity threshold—500. After checking the regions, all *m*/*z* points were annotated by the following libraries: HMDB library 2.0_KEGG, Lipids Human Brain metabolites library, Lipids Mouse Kidney metabolites library, Small Molecules metabolites library, N-Glycan human library, Cell culture nutrient library, Fatty acids library, HMDB plasma metabolites library, Lipid maps library, Natural products metabolites library, and the CCS compendium library. Data were additionally annotated by using a range of mass spectral libraries provided by Metaboscape, such as Bruker Sumner MetaboBASE plant library, Bruker NIST 2020 MSMS Spectral Library hr-2, and MSDIAL-Tandem Mass Spectral Atlas libraries for both positive and negative ions. The parameters (tolerances and scoring) used for annotations were as follows *m*/*z*: 2.0–5.0 ppm, mSigma 25–500, and CCS 2.0–5.0%. Annotations of metabolites against all Lipid classes that were available in Metaboscape were also carried out with the same *m*/*z* and mSigma values.

### 4.7. Statistical Analysis

Bucket tables from each experiment were exported from Metaboscape 2021b for further statistical data analysis in R. Nonmetric dimensional scaling was carried out in R by using the vegan package. To calculate the statistical difference between the concentration of a metabolite in the groups tested, ANOSIM was employed. For heatmap generation, *p*-values (FDR) were calculated by t-tests between the two groups, and we plotted a heatmap of the top 50 most significantly different metabolites between the two groups. Heatmap library was used to plot the heatmap. After the peak intensity table was imported, the uploaded data were log-transformed, and normalization was performed by mean subtraction. Other parameters that were set included the use of correlation-based clustering of the columns. To simplify the visualization of the abundances of metabolites across the treatments, the top 50 metabolites ranked by t-test were shown. To visualize the shared and unique metabolites between the two groups, a Venn diagram was generated using the Bioinformatics and Evolutionary Genomics Venn diagram tool. (https://bioinformatics.psb.ugent.be/webtools/Venn/, accessed on 22 December 2021).

### 4.8. Pathway Analysis

*m*/*z* values of all metabolites were ranked based on the *p*-value (FDR) obtained after carrying out the pairwise comparison between groups. These *p*-values were used for pathway analysis using the functional analysis option in MetaboAnalyst (version 5, USA). Positive ion mode with a mass tolerance of 5 ppm was selected for data processing. After passing the filtering parameters, pathways were elucidated by using *Mus musculus* (mouse) (KEGG library). Only those pathways having at least 3 metabolite entries were considered.

### 4.9. Immunohistochemistry of Fresh Frozen Tissue

After MALDI imaging, the same tissue samples were used for IHC staining. To remove the matrix from tissue samples, and slides were placed in absolute ethanol for 10 min, followed by a 15 min incubation in the 1x PBS solution to remove the OCT compound. Both FFPE and fresh frozen tissue were treated the same. They were incubated in 95% ethanol for 5 min, 70% ethanol for 5 min, and 50% ethanol for 5 min. The slides were then rinsed with distilled water and placed in 1× PBS for 10 min. Fresh frozen tissue was fixed with 4% PFA for 30 min at room temperature and then washed three times in PBS. Subsequently, both the FFPE and fresh frozen tissue samples were incubated in a solution containing 0.5% Triton X-100, Alexa-Flour Phalloidin-488, and DAPI for 30 min at room temperature. The samples were then washed three times with PBS, with fifteen minutes between washes. Samples were mounted with gold antifade mounting medium (# P36930 Invitrogen, Life Technologies Corporation, Eugene, OR, USA). Slides were imaged with a Cytation 5 microscope at 4× and 20× magnification.

## Data Availability

The data presented in this study are available in Supplementary Material.

## Supplementary Materials

The following supporting information can be downloaded at: https://www.mdpi.com/article/10.3390/metabo12060497/s1, Table S1: FFPE vs Fresh Frozen +ve ion mode, Table S2: Fresh Frozen vs FFPE +ve ion mode, Table S3: Common metabolites +ve ion mode, Table S4: FFPE vs Fresh Frozen -ve ion mode, Table S5: Fresh Frozen vs FFPE -ve ion mode, Table S6: Common metabolites -ve ion mode.
