# Supplementary material for: Impact of Skin Tissue Collection Method on Downstream MALDI-Imaging
Source: Metabolites. 2022 May 30;12(6):497. doi: 10.3390/metabo12060497 (PMC9227925; doi:10.3390/metabo12060497)
Supplement: Supplementary file 1 [file metabolites-12-00497-s001.zip › metabolites-1672290-supplementary.pdf]

Impact of skin tissue collection method on downstream MALDI imaging

Manoj Yadav[1,*,¥], Prem Prashant Chaudhary[1,*], Brandon D'Souza[1], Jacquelyn Spathies[1], and Ian A Myles[1]

1 – Epithelial Therapeutics Unit, National Institute of Allergy and Infectious Disease, National Institutes of Health, Bethesda, MD, USA

* Author contributed equally

¥Corresponding author: manoj.yadav@nih.gov

Supplementary Table S1

**FFPE vs Fresh Frozen +ve ion mode**
Purine metabolism
Pyrimidine metabolism
N-Glycan biosynthesis
Glycosphingolipid biosynthesis - globo and isoglobo series
Glycosphingolipid biosynthesis - lacto and neolacto series
Fructose and mannose metabolism
Fatty acid biosynthesis
Fatty acid elongation
Amino sugar and nucleotide sugar metabolism
Metabolism of xenobiotics by cytochrome P450
Drug metabolism - other enzymes

Supplementary Table S2

**Fresh Frozen vs FFPE +ve ion mode**
Glycosaminoglycan degradation
Glycosphingolipid biosynthesis - lacto and neolacto series
N-Glycan biosynthesis
Steroid biosynthesis
Primary bile acid biosynthesis
Mucin type O-glycan biosynthesis
Mannose type O-glycan biosynthesis
Glycosphingolipid biosynthesis - globo and isoglobo series
Biosynthesis of unsaturated fatty acids

Supplementary Table S3

**Common metabolites +ve ion mode**
Steroid hormone biosynthesis
Purine metabolism
Pyrimidine metabolism
Amino sugar and nucleotide sugar metabolism
Glycosylphosphatidylinositol (GPI)-anchor biosynthesis
Glycerophospholipid metabolism
One carbon pool by folate
Riboflavin metabolism
Nicotinate and nicotinamide metabolism

Porphyrin and chlorophyll metabolism

Supplementary Table S4

**FFPE vs Fresh Frozen -ve ion mode**
Glycosphingolipid biosynthesis - lacto and neolacto series
N-Glycan biosynthesis
Primary bile acid biosynthesis
Various types of N-glycan biosynthesis
Inositol phosphate metabolism
Glycosphingolipid biosynthesis - globo and isoglobo series
Folate biosynthesis
Porphyrin and chlorophyll metabolism
Biosynthesis of unsaturated fatty acids
Phosphatidylinositol signaling system

Supplementary Table S5

**Fresh Frozen vs FFPE -ve ion mode**
Purine metabolism
Folate biosynthesis
Riboflavin metabolism
Pyrimidine metabolism
Mannose type O-glycan biosynthesis
Drug metabolism - other enzymes
Citrate cycle (TCA cycle)
Pentose phosphate pathway
Pentose and glucuronate interconversions
Fructose and mannose metabolism
Alanine, aspartate and glutamate metabolism
Phosphonate and phosphinate metabolism
Amino sugar and nucleotide sugar metabolism
Glycerophospholipid metabolism
Pyruvate metabolism
One carbon pool by folate
Pantothenate and CoA biosynthesis
Porphyrin and chlorophyll metabolism
Terpenoid backbone biosynthesis
Metabolism of xenobiotics by cytochrome P450
Drug metabolism - cytochrome P450

Supplementary Table S6

**Common metabolites -ve ion mode**
Fatty acid degradation
Butanoate metabolism
Terpenoid backbone biosynthesis
Citrate cycle (TCA cycle)
Pantothenate and CoA biosynthesis
Pyrimidine metabolism
Amino sugar and nucleotide sugar metabolism
Metabolism of xenobiotics by cytochrome P450
Glycerophospholipid metabolism
Propanoate metabolism
Valine, leucine and isoleucine degradation
Drug metabolism - other enzymes
Purine metabolism
Pentose and glucuronate interconversions
Galactose metabolism
Ascorbate and aldarate metabolism
Synthesis and degradation of ketone bodies
Alanine, aspartate and glutamate metabolism
Cysteine and methionine metabolism
Valine, leucine and isoleucine biosynthesis
Lysine degradation
Tryptophan metabolism
Phosphonate and phosphinate metabolism
Starch and sucrose metabolism
Glycerolipid metabolism
Pyruvate metabolism
Glyoxylate and dicarboxylate metabolism
Drug metabolism - cytochrome P450
